# Effects of statin therapy on chronic kidney disease patients with coronary artery disease

**DOI:** 10.1186/s12944-018-0742-4

**Published:** 2018-04-17

**Authors:** Hao Shen, Xiaodong Chen, Jingfen Lu, Honglin Yang, Yan Xu, Ao Zhu, Xiao Zhang, Fulong Ye, Yongchun Gu

**Affiliations:** 1 Department of Clinical Laboratory Medicine, First People’s Hospital of Wujiang District, Nantong University, Suzhou, China; 2 Department of Internal Medicine, First People’s Hospital of Wujiang District, Nantong University, Suzhou, China; 3 Department of Nephrology, First People’s Hospital of Wujiang District, Nantong University, Suzhou, China; 4 Department of Central Laboratory, First People’s Hospital of Wujiang District, Nantong University, Suzhou, China

**Keywords:** Chronic kidney disease, Statin, Cardiovascular events

## Abstract

**Background:**

Long-term persistence of statin therapy provided an ongoing reduction in mortality among patients with and without a known history of CVD, and renoprotective effect on CKD patients. Until now, very few reports are available from China to address the effects of statin therapy in CKD + CAD patients.

**Methods:**

We compared the effects of long-term statin therapy (follow-up time 4 years) in terms of cardiovascular events, all-cause death, and cardiac death among 254 CKD patients with or without CAD.

**Results:**

Long-term statin therapy was much more effective in the CKD + CAD patients compared with CKD patients. In the CAD + CKD patients, long-term statins showed a 22.2% reduction in the CVs rate (*P* = 0.012). With regard to the all-cause and cardiac deaths, long-term statins had significant treatment effects on the CAD + CKD patients (reduction of about 28.1% in mortality rates, *P* < 0.001). In contrast, long-term statin therapy exerted no significant influence on the clinical outcomes of the CKD-only patients.

**Conclusion:**

Long-term statin therapy more dramatically reduced the CVs and mortality rates of the CKD patients with concomitant CAD. In contrast, CKD-only patients had a good prognosis and did not appear to require statin treatment.

## Background

Chronic kidney disease (CKD) is increasing worldwide, it has been recognized as a major and independent risk factor for cardiovascular events (CVs) [1–3], such as coronary artery disease (CAD). Patients with CAD were frequently shown to have impaired renal function, which may contribute to worse clinical outcomes. On the other hand, most CKD patients die of cardiovascular diseases (CVD) than to progress to end stage kidney disease [4–6].

Statins, the 3-hydroxy 3-methyl-glutaryl coenzyme-A reductase inhibitors,that are well tolerated and the first-choice agents for low-density lipoprotein cholesterol (LDL-C) reduction. They are reported to reduce CVs, including myocardial infarction, stroke, and death [7–9]. In patients with mild-to-moderate CKD, statins were found to be effective for primary prevention and to reduce cardiovascular risk [10–14]. Thus, it is believed that statins have both cardiovascular and renal benefits in prevention and treatment.

Long-term persistence of statin therapy provided an ongoing reduction in mortality among patients with and without a known history of CVD, and renoprotective effect on CKD patients [15, 16]. Until now, very few reports are available from China to address the effects of statin therapy in CKD + CAD patients. Consequently, we compare the effects of long-term statin therapy on CVs and mortality in Chinese CKD patients with or without CAD.

## Methods

### Study participants

Hospitalization records of 254 patients with CKD (stage3–4) + CAD, or CKD (stage3–4) only were reviewed for demographic data, cardiovascular end points, and long-term regular use of statins. Inpatients were selected from the Wujiang Affiliated Hospital of Nantong University between December, 2010 and December, 2012. Of these, 128 CKD patients were considered to have concomitant CAD if they were diagnosed with CAD by coronary angiogram (CAG), patients received a stent graft or coronary artery bypass grafting surgery according to the extent of their lesions, and 126 CKD-only patients were randomly selected for the control groups. We used a simplified MDRD equation, which is more suitable for Chinese people, to evaluate kidney functions: 186 × (serum creatinine)^− 1.154^ × Age^− 0.203^ × (0.742 for female). The staging of renal function was categorized according to the K/DOQI guidelines [17, 18]: stage 3 is a moderate reduction in eGFR (30–59 mL/min/1.73 m^2^), stage 4 is a severe reduction in eGFR (15–29 mL/min/1.73 m^2^).

Hospitalization records of the 254 patients were reviewed for cardiovascular end points: The primary end points (death, including cardiovascular and non-cardiovascular mortality), secondary end points (revascularization, angina, nonfatal myocardial infarction, heart failure, ischemic stroke, and hemorrhagic stroke), Long-term statin users were defined: patients who had taken medication not less than 50% of the follow-up time minus 30 days to the end of follow-up [19]. Otherwise, the patients were categorized into the no-statin group, and demographic data, included gender, age, body mass index (BMI), history of hypertension and diabetes mellitus [20]. Acute myocardial infarction, angina, and heart failure were diagnosed according to the 2008 European Society of Cardiology guidelines. Stroke was diagnosed by the 2010 Chinese guidelines for diagnosis and treatment of acute ischemic stroke [21].

Exclusion criteria included diagnosis of CAD prior to admission; long-term treatment with lipid-lowering drugs prior to admission; missing blood examination data; patients who died during the hospitalization; patients suffering from major diseases, and expected to survive less than 3 months.

### Laboratory measurements

Various biochemical indexes: fasting blood glucose (FBG), triglyceride (TG), total cholesterol (TC), high-density lipoprotein cholesterol (HDL-C), LDL-C, lipoprotein (a) (Lp-a), apolipoprotein A-I (ApoA-I), apolipoprotein B (ApoB), and glycated hemoglobin (HbA1C) were measured via HPLC method [20].

### Statistical analyses

Continuous and categorical data were presented as mean ± standard deviation (SD) and number (%), t-test for parametric variables and Wilcoxon tests for non-parametric variables were used to assess group differences (CKD vs CKD + CAD). Comparisons of categorical variables were performed by the Chi-square test. The long-term event-free rate was estimated using Kaplan-Meier curves, and the log-rank test was used to identify significant differences in survival rates between the two groups. A univariate Cox regression model was used to identify the potential factors that might affect the incidence of cardiovascular events. Furthermore, a multivariate Cox regression analysis was developed to determine those independent predictors. Hazard ratios and corresponding 95% confidence intervals were sued to describe the intensity for the risk of cardiovascular events. All the statistical analyses were two-tailed. And *P* < 0.05 was considered statistically significant. All statistical analyses were performed using SAS 9.1(SAS Institute, Cary, NC, USA).

## Results

### Patients^,^Characteristics

A total of 254 patients were examined in this research. The CAD + CKD group included 128 patients. The CKD-only group had 126 patients, respectively. Table 1 summarizes the characteristics of patients in the two groups. The oldest patients and Male gender was found more often in the CKD + CAD group. It is noteworthy that Hypertension, myocardial infarction, smoking, and angina pectoris were more common in the CKD + CAD group. There were significant differences in statin therapy: 90.6% of CKD + CAD patients took statins compared with only 46.0% of CKD patients. There was also a significant difference in other medications therapy between the two groups, such as aspirin, β-blockers, calcium-channel blockers, angiotensin-converting enzyme inhibitors (ACEI) and angiotensin-receptor blockers (ARB). Table 2 shows the laboratory characteristics of the subjects. There was a significant difference in TG, TC, LDL-C, HDL-C, Glucose, HbA1c and DBP at baseline between the two groups, and the other parameters in the baseline characteristics were similar.

**Table 1:** The clinical characteristics at baseline

Variables	CKD + CAD	CKD	*P* ^a^
	(*n* = 128)	(*n* = 126)	
Characteristics			
Age (years)	69.7 ± 8.4	53.6 ± 17.8	< 0.001
Male	90/128 (70.3)	54/126 (42.8)	< 0.001
BMI (kg/m^2^)	24.3 ± 2.1	23.4 ± 2.2	0.001
Hypertension	95/128 (74.2)	71/126 (56.3)	0.003
Diabetes mellitus	34/128 (26.5)	36/126 (28.5)	0.72
Smoking	58/128 (45.3)	21/126 (16.6)	< 0.001
Myocardial infarction	72/128 (56.2)	0/126 (0)	< 0.001
Angina pectoris	51/128 (39.8)	0/126 (0)	< 0.001
Stroke	7/128 (5.4)	6/126 (4.7)	0.798
Medications			
Calcium-channel blocker	45/128 (35.1)	64/126 (50.7)	0.012
ACEI	57/128 (44.5)	23/126 (18.2)	< 0.001
ARB	46/128 (35.9)	66/126 (52.3)	0.008
*β*-blocker	92/128 (71.8)	37/126 (28.6)	< 0.001
Loop diuretic	19/128 (14.8)	11/126 (8.7)	0.131
Aspirin	117/128 (91.4)	37/126 (29.3)	< 0.001
Statin	116/128 (90.6)	58/126 (46.0)	< 0.001

Data are presented as mean ± SD or the number and its percentage (%). Percentage = the number of each individual category divided by n

*CAD* coronary artery disease, *CKD* chronic kidney disease, *ACEI* angiotensin-converting enzyme inhibitor, *ARB* angiotensin-receptor blocker, *BMI* body mass index

^a^ indicates the comparison of mean or percentage between CKD + CAD group and CKD group

**Table 2:** The laboratory characteristics at baseline

Variables	CKD + CAD	CKD	*P* ^a^
	(*n* = 128)	(*n* = 126)	
Triglycerides (mmol/l)	1.7 ± 1.1	2.5 ± 1.3	< 0.001
Total cholesterol (mmol/l)	4.8 ± 1.3	6.6 ± 2.8	< 0.001
LDL-C (mmol/l)	6.7 ± 1.9	4.1 ± 2.4	< 0.001
HDL-C (mmol/l)	1.0 ± 0.4	1.3 ± 0.4	< 0.001
Glucose (mmol/l)	7.3 ± 4.4	9.1 ± 5.2	0.003
HbA1c (%)	6.5 ± 1.2	7.9 ± 2.3	< 0.001
SBP (mmHg)	137.1 ± 31.2	139.3 ± 21.9	0.517
DBP (mmHg)	78.3 ± 15.1	73.1 ± 10.2	0.001
ApoA-I (g/l)	1.3 ± 2.8	1.3 ± 0.3	0.999
ApoB (g/l)	1.0 ± 2.2	1.0 ± 0.3	0.999
ApoA-I/B	1.3 ± 0.3	1.4 ± 0.5	0.054
Lp (a) (ng/ml)	382.3 ± 326.4	373.4 ± 394.2	0.845

Data are presented as mean ± SD

*CAD* coronary artery disease, *CKD* chronic kidney disease, *HbA1c* hemoglobin A1c, *ApoA-I* apolipoprotein A-I, *ApoB* apolipoprotein B, *HDL-C* high-density lipoprotein cholesterol, *LDL-C* low-density lipoprotein cholesterol, *SBP* systolic blood pressure, *DBP* diastolic blood pressure, *Lp (a)* lipoprotein (a)

^a^ indicates the comparison of mean between CKD + CAD group and CKD group

### Clinical outcomes

**Fig. 1:**
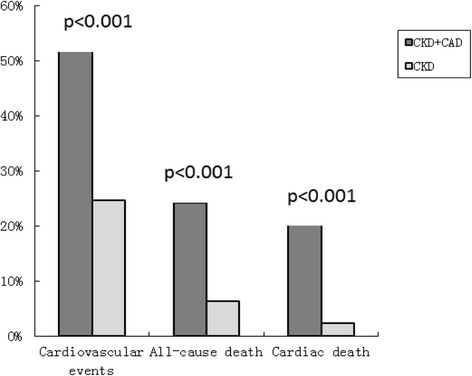
Clinical outcomes in CKD + CAD and CKD groups

**Fig. 2:**
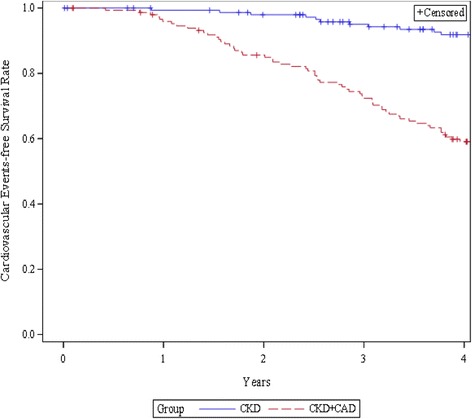
Kaplan-Meier curves for cardiovascular events free survival rates for the two groups

The CKD + CAD group was found to have CVs (54.6%), all-cause death (24.2%), and cardiac death (20.3%) with the higher frequency among the two groups (Fig. 1). The incidence of CVs was lower in the CKD-only group (24.5%) compared with that of the CKD + CAD group (*P* < 0.001). Figure 2 shows the cardiovascular events-free survival curves through 4 years for the two groups. The log-rank test revealed that the cardiovascular events -free survival rate in the CAD + CKD group was lower compared with that of the CKD group (P < 0.001).

### Effects of statins on clinical outcomes

We compared the effects of statins on the clinical outcomes of the two groups of subjects. The data of Table 3 revealed that long-term statin therapy was much more effective in the CKD + CAD patients compared with CKD patients. In the CAD + CKD patients, long-term statins showed a 22.2% reduction in the CVs rate (*P* = 0.012). With regard to the all-cause and cardiac deaths, long-term statins had significant treatment effects on the CAD + CKD patients (reduction of about 28.1% in mortality rates, P < 0.001). In contrast, long-term statin therapy exerted no significant influence on the clinical outcomes of the CKD-only patients.

**Table 3:** Effects of statins on clinical outcomes

Variables	Long-term statins	No statin	*P* ^a^
CKD + CAD			
Cardiovascular events	29/66 (43.9%)	41/62 (66.1%)	0.012
All-cause death	7/66 (10.6%)	24/62 (38.7%)	< 0.001
Cardiac death	5/66(7.5%)	21/62 (33.8%)	< 0.001
CKD			
Cardiovascular events	12/35 (34.2%)	19/91 (20.8%)	0.118
All-cause death	2/35 (5.7%)	6/91 (6.5%)	0.856
Cardiac death	0/35 (0%)	3/91 (3.2%)	0.56

Data are presented as the number and its percentage (%)

*CAD* coronary artery disease, *CKD* chronic kidney disease

^a^ indicates the comparison of percentage between Long-term statins group and No statin group

### Predictors for cardiovascular events

Univariate Cox regression analysis showed that group, age, hypertension, smoking, myocardial infarction, β-blockers, aspirin, LDL-C, statin discontinuation at final observation might affect the incidence of CVs.

Multivariate Cox regression analysis, including the significant predictors above and the marginally significant predictor of myocardial infarction in the univariate model, showed that group, smoking, LDL-C, statin discontinuation were independent predictors of CVs (Table 4). The CKD-only group had the lower risk of CVs (versus CAD + CKD group: HR 0.46, 95% CI 0.25–0.59, *P* < 0.001. The findings indicate that the CKD group had the better long-term survival status than CAD + CKD group.

**Table 4:** Predictors for Cardiovascular events according to Cox’s proportional hazard analysis

Variables		Unadjusted			Adjusted	
	HR	95% CI	*P*	HR	95% CI	*P*
CKD versus CKD + CAD	0.5	(0.27–0.68)	< 0.001	0.46	(0.25–0.59)	< 0.001
Age (years)	1.32	(1.21–1.93)	< 0.001	1.1	(0.95–1.04)	0.078
Male (versus female)	0.76	(0.57–1.15)	0.545		–	
Hypertension	1.61	(1.21–2.01)	< 0.01	1.16	(0.95–1.67)	0.071
Diabetes mellitus	1.24	(0.87–1.72)	0.291		–	
Smoking	1.43	(1.33–1.81)	< 0.01	1.41	(1.09–2.11)	0.009
Myocardial infarction	1.18	(1.06–1.89)	0.048	1.03	(0.91–1.35)	0.121
Angina pectoris	1.07	(0.67–1.46)	0.32		–	
Stroke	1.53	(0.97–3.16)	0.221		–	
Calcium-channel blocker	1.27	(0.76–1.50)	0.322		–	
ACEI	0.97	(0.85–1.30)	0.976		–	
ARB	0.89	(0.63–1.28)	0.514		–	
*β*-blocker	1.42	(1.12–1.89)	< 0.01	1.11	(0.72–1.61)	0.55
Loop diuretic	1.09	(0.61–1.76)	0.488		–	
Aspirin	1.98	(1.56–3.97)	< 0.01	1.32	(0.68–1.99)	0.601
Triglycerides (mmol/l)	1.12	(0.72–1.81)	0.78		–	
Total cholesterol (mmol/l)	1.07	(0.87–1.75)	0.64		–	
LDL-C (mmol/l)	1.12	(1.07–1.62)	< 0.001	1.05	(1.01–1.25)	0.011
HDL-C (mmol/l)	0.69	(0.46–1.21)	0.222		–	
Glucose (mmol/l)	1.23	(0.96–1.91)	0.52		–	
HbA1c (%)	0.96	(0.68–1.69)	0.771		–	
SBP (mmHg)	0.97	(0.90–1.18)	0.078		–	
DBP (mmHg)	1.08	(0.97–1.39)	0.56		–	
Long-term statins	0.22	(0.19–0.57)	< 0.001	0.27	(0.21–0.41)	< 0.001

*CAD* coronary artery disease, *CKD* chronic kidney disease, *ACEI* angiotensin-converting enzyme inhibitor, *ARB* angiotensin-receptor blocker, *SBP* systolic blood pressure, *DBP* diastolic blood pressure, *HbA1c* hemoglobin A1c, *HDL-C* high-density lipoprotein cholesterol, *LDL-C* low-density lipoprotein cholesterol

## Discussion

Our study compared the effects of long-term statin treatment in CKD and CAD + CKD patients in China. In the current study, we show that, among the Chinese CKD patients, the rate of CAD was as high as 50.4% (128/254). In particular, the all-cause and cardiac death rates of the CAD + CKD patients were about twice as high as the CKD-only patients. Thus, just as CKD is a risk factor for CAD, CAD is a risk factor for CKD, emphasizing that CAD and CKD are closely associated diseases with a reciprocal adverse impact. Our data show that long-term statin therapy can significantly improve the clinical outcomes of the CAD + CKD patients, the treatment effect of statins was much more dramatic, with remarkable reductions in the CVs rate (22.2%), all-cause death (28.1%), and cardiac death (26.3%). In contrast, no significant treatment effect of statin was observed in the CKD-only patients. In the current study, the CAD + CKD patients had a high rate of hypertension, smoking, myocardial infarction, and angina pectoris. The unadjusted determinants of CVs included hypertension, smoking, myocardial infarction, LDL-C level, and the use of β-blockers and aspirin. After adjustment, smoking, statin therapy, LDL-C level were significant predictors of CVs. These findings are consistent with previous reports in general [5, 6, 22].

To our knowledge,few studies have compared the effects of long-term statin therapy in the CKD patients with or without CAD from China. Some study [23, 24] compared the effects of statins on the clinical outcomes of CAD patients with or without renal insufficiency; however, there was no CKD-only patient group in their investigation. Longer-term statin therapy may exert more significant treatment effects, and our results show that the extent of reduction in CVs, all-cause deaths, and cardiac deaths appears to be more dramatic compared with the Japanese studies. This suggests that the Chinese CKD + CAD patients are substantially different from those in Japan. Possible reasons for the high morbidity and mortality in China include worse economic conditions, heavier labor, and insufficient medical care. Thus, in CKD + CAD patients with worse clinical outcomes like in China, long-term statin therapy can exert more dramatic treatment effects, and measures should be taken to enhance adherence to statins.

One striking discovery is that the CKD-only patients had very low all-cause or cardiac mortality, and statin therapy did not have a significant impact on the CVs and mortality rates. Statin treatment has been shown to be beneficial for CKD patients not undergoing dialysis [10, 16, 25]; however, previous studies have not separated CKD patients with or without CVD. In the current study, we selectively analyzed the CKD patients who did not have CVD at registration; these patients did not develop significantly increased CVs and mortality rates during the long follow-up period nor did they show an effective response to statin treatment. Thus, CKD patients free of CVD at baseline appear to have an excellent prognosis and, therefore, may not need statin treatment. This finding may provide a significant guideline regarding statins in CKD patients, and it warrants larger scale studies across multiple clinical centers in China.

There were several limitations in this study. Firstly, this is not a prospective study. Therefore, we need a more carefully controlled prospective study to achieve this purpose in the future. Secondly, the sample size of this study was small. Therefore, future studies that include a larger cohort of patients are necessary to corroborate our findings.

## Conclusion

This study compared CKD patients with or without CAD treated with long-term statins; it provides new insights about the appropriate treatment of these two groups of patients. Long-term statin therapy more dramatically reduced the CVs and mortality rates of the CKD patients with concomitant CAD. In contrast, CKD-only patients had a good prognosis and did not appear to require statin treatment.

## Abbreviations

Apo: Apolipoprotein
BMI: Body mass index
CAD: Coronary artery disease
CKD: Chronic kidney disease
CVD: Cardiovascular diseases
CVs: Cardiovascular events
DBP: Diastolic blood pressure
eGFR: Estimated glomerular filtration rate
FBG: Fasting blood glucose
HbAlc: Hemoglobin A1c
HDL-C: High-density lipoprotein cholesterol
LDL-C: Low-density lipoprotein cholesterol
SBP: Systolic blood pressure
TC: Total cholesterol
TG: Triglyceride
